# The Protective Effects of an Aged Black Garlic Water Extract on the Prostate

**DOI:** 10.3390/nu16173025

**Published:** 2024-09-07

**Authors:** Maria Loreta Libero, Antonio J. Montero-Hidalgo, Lucia Recinella, Raúl M. Luque, Daniele Generali, Alessandra Acquaviva, Giustino Orlando, Claudio Ferrante, Luigi Menghini, Simonetta Cristina Di Simone, Nilofar Nilofar, Annalisa Chiavaroli, Luigi Brunetti, Sheila Leone

**Affiliations:** 1 Department of Pharmacy, “G. d’Annunzio” University, 66013 Chieti, Italy; maria.libero@unich.it (M.L.L.); alessandra.acquaviva@unich.it (A.A.); giustino.orlando@unich.it (G.O.); claudio.ferrante@unich.it (C.F.); luigi.menghini@unich.it (L.M.); simonetta.disimone@unich.it (S.C.D.S.); nilofar.nilofar@unich.it (N.N.); annalisa.chiavaroli@unich.it (A.C.); luigi.brunetti@unich.it (L.B.); sheila.leone@unich.it (S.L.); 2 Department of Cell Biology, Physiology and Immunology, University of Cordoba, 14014 Cordoba, Spain; b42mohia@uco.es; 3 Maimonides Biomedical Research Institute of Cordoba (IMIBIC), 14004 Cordoba, Spain; 4 Reina Sofia University Hospital (HURS), 14004 Cordoba, Spain; 5 Centro de Investigación Biomédica en Red de Fisiopatología de la Obesidad y Nutrición (CIBERobn), 14004 Cordoba, Spain; 6 Department of Medical, Surgical and Health Sciences, University of Trieste, 34149 Trieste, Italy; dgenerali@units.it; 7 Department of Advanced Translational Microbiology, Institute for Maternal and Child Health-IRCCS “Burlo Garofolo”, 34137 Trieste, Italy

**Keywords:** ABGE, prostate, inflammation, cancer

## Abstract

Chronic inflammation is a recognized risk factor for various cancers, including prostate cancer (PCa). We aim to explore the potential protective effects of aged black garlic extract (ABGE) against inflammation-induced prostate damage and its impact on prostate cancer cell lines. We used an ex vivo model of inflammation induced by Escherichia coli lipopolysaccharide (LPS) on C57BL/6 male mouse prostate specimens to investigate the anti-inflammatory properties of ABGE. The gene expression levels of pro-inflammatory biomarkers (*COX-2*, *NF-κB*, and *TNF-α*, *IL-6*) were measured. Additionally, we evaluated ABGE’s therapeutic effects on the prostate cancer cell lines through in vitro functional assays, including colony formation, tumorsphere formation, migration assays, and phosphorylation arrays to assess the signaling pathways (MAPK, AKT, JAK/STAT, and TGF-β). ABGE demonstrated significant anti-inflammatory and antioxidant effects in preclinical models, partly attributed to its polyphenolic content, notably catechin and gallic acid. In the ex vivo model, ABGE reduced the gene expression levels of *COX-2*, *NF-κB*, *TNF-α*, and *IL-6*. The in vitro studies showed that ABGE inhibited cell proliferation, colony and tumorsphere formation, and cell migration in the prostate cancer cells, suggesting its potential as a therapeutic agent. ABGE exhibits promising anti-inflammatory and anti-cancer properties, supporting further investigation into ABGE as a potential agent for managing inflammation and prostate cancer.

## 1. Introduction

Prostatitis is a prostate gland inflammation, which encompasses a range of disorders, such as acute and chronic bacterial prostatitis. These conditions can be caused by bacterial infections, immune responses, or non-infectious factors such as trauma or stress [1].

In particular, acute prostatic inflammation in mice induced an epithelial transformation, named proliferative inflammatory atrophy, which could promote prostatic intraepithelial neoplasia [1].

In this context, chronic inflammation is often linked with the process of carcinogenesis and is recognized as both a hallmark and a potential risk factor for various cancers [2]. Specifically, for prostate cancer (PCa), chronic inflammation is suggested as a bridge between environmental factors and tumor development [3,4,5].

Numerous studies have explored the relationship between prostate gland abnormalities and the inflammatory process, showing a strong prevalence of mild chronic inflammation in PCa [6].

Chronic inflammation can create a microenvironment conducive to carcinogenesis by producing pro-inflammatory cytokines, reactive oxygen species, and DNA damage [7].

In line with this, NLRP3 inflammasome is critically involved in PCa aggressiveness [8].

Altogether, the presented evidence indicates the proficient pro-oncogenic role of certain inflammatory processes in PCa [9,10].

Various biomarkers, such as tumor necrosis factor (TNF)-α, nuclear factor (NF)-kB, interleukin (IL)-6, and cyclooxygenase (COX)-2, play a critical role in inflammatory responses. In particular, Baud and their collaborators (2001) reported that TNF-α is a potent pro-inflammatory cytokine whose involvement in inflammation, cell proliferation, differentiation, and apoptosis is well known. Increased serum levels of pro-inflammatory markers such as TNF-α are related to accelerated progression and a poor prognosis in PCa [11,12].

Furthermore, NF-κB is essential for regulating both the innate and adaptive immune responses, particularly in inflammation. Besides its role in the survival and activation of immune cells, NF-κB stimulates the release of pro-inflammatory genes, including cytokines and chemokines, and regulates inflammasome activity. Moreover, the dysregulation of NF-κB contributes to various inflammatory diseases, including rheumatic diseases and asthma [13,14]. Interestingly, a wide body of evidence suggested that NF-κB activation, as well as various signals linked to inflammation, are well known to be involved in the modulation of PCa malignancy [15]. In particular, NF-κB activation exerts modulatory effects on the expression of the cytokines and factors involved in cancer development and progression, including IL-6 [15]. Moreover, the activation of IL-6 signaling was found to induce growth, proliferative activity, and the migration of PCa cells [16].

COX-2 is also critically involved in carcinogenesis in various tissues, including breasts and lungs, as well as the prostate [17].

Various studies suggested the potential activity of a number of herbal extracts commonly used in traditional medicine as well as natural compounds exhibiting an innovative action mode as a possible remedy for PCa [18,19].

In this context, aged black garlic (ABG) has garnered attention for its bioactive compound profile and biological activities [20].

ABG is produced by fermenting fresh garlic at controlled high humidity (80–90%) and temperature (60–90 °C) conditions over several weeks. As previously reported [20], the temperature and humidity conditions of the thermal treatment chosen during ABG production are strongly involved in the quality of ABG. 

This process alters garlic’s organoleptic properties, making it sweeter and less pungent, and increases the concentration of bioactive compounds, such as S-allylcysteine, polyphenols, and flavonoids [21]. These compounds were found to be able to exert various beneficial effects, including the suppression of cell proliferative activity, as well as the stimulation of apoptosis and the modulation of the cell cycle, all of which are relevant in cancer prevention and treatment [22,23].

Interestingly, in the previous studies of ours, a water extract of ABG (ABGE) showed anti-inflammatory and antioxidant effects in preclinical models [24,25]. In particular, the protective effects induced by ABGE were suggested to be partly related to the polyphenolic content in the same extract, notably catechin and gallic acid [24,25]. We previously performed the quantification of polyphenolic content in the extract using high-performance liquid chromatography coupled with a photo diode array detector (HPLC-DAD) analytical method. In particular, various compounds were identified in ABGE, with gallic acid and catechin being the more representative phytochemicals [26].

This research aims to explore the potential benefits of ABGE on inflammation and prostate cancer. Building on these findings, we sought to explore the potential protective effects of ABGE against inflammation-induced prostate damage using an ex vivo experimental model, as well as its impact on prostate cancer cell lines through in vitro studies. We investigated the anti-inflammatory properties of ABGE using a well-established ex vivo model of inflammation composed of mouse prostate specimens exposed to *Escherichia coli* lipopolysaccharide (LPS) [26,27]. In this setting, we examined the gene expression levels of the key pro-inflammatory biomarkers, including *COX-2*, *NF-κB*, *TNF-α*, and *IL-6*. Furthermore, we assessed the potential therapeutic effects of ABGE on the prostate cancer cell lines using in vitro experimental models through functional parameters (colony formation, tumorsphere formation, and a migration assay) and molecular studies to evaluate the potential involvement of different signaling pathways, such as mitogen-activated protein kinase (MAPK), protein kinase B (AKT), Janus kinases/the signal transducer and activator of transcription proteins (JAK/STAT), and transforming growth factor (TGF-β).

## 2. Materials and Methods

### 2.1. Extraction and Sample Preparation of ABGE

Dried ABG cloves were provided by il Grappolo S.r.l. (Soliera, Modena, Italy). The preparation of ABGE followed the method described in the previous studies [24,28,29].

A detailed protocol is included in the Supplementary Materials.

### 2.2. Ex Vivo Studies

Adult C57BL/6 male mice (3 months old, weight 20–25 g, n = 25) were housed and maintained as described in the Supplementary Materials Section. The housing conditions and experimentation procedures were strictly in agreement with the European Community ethical regulations (EU Directive no. 26/2014) for the care of animals for scientific research. In agreement with the recognized principles of “Replacement, Refinement and Reduction in Animals in Research”, prostate specimens were obtained as residual materials from the vehicle-treated mice randomized in our previous experiments, approved by the local ethical committee (‘G. d’Annunzio’ University, Chieti, Italy) and the Italian Health Ministry (Project no. 885/2018-PR).

Mouse sacrifice was performed by CO_2_ inhalation (100% CO_2_ at a flow rate of 20% of the chamber volume per min). After collection, the isolated prostate specimens were maintained in a humidified incubator with 5% CO_2_ at 37 °C for 4 h, as previously described [26,30] and reported in the Supplementary Materials Section.

Total RNA was extracted from the prostate specimens using TRI reagent (Sigma-Aldrich, St. Louis, MO, USA) following the manufacturer’s protocol. The gene expression of COX-2, NF-kB, TNF-α, and iNOS was quantified by real-time PCR with TaqMan probe-based chemistry, as previously described [27,31,32]. The detailed protocol can be found in the Supplementary Materials Section.

### 2.3. Cell Culture

Cell lines from control prostate (PNT-2), androgen-dependent PCa (LNCaP), and androgen-independent PCa (PC-3) (American Type Culture Collection, Manassas, VA, USA) were maintained in a humidified incubator with 5% CO_2_ at 37 °C following the manufacturer’s guidelines as previously outlined [33,34] (Supplementary Materials Section).

### 2.4. Cell Proliferation

Cell proliferation was evaluated using resazurin reagent (Canvax Biotech, Cordoba, Spain) [33]. Cell proliferation was measured at the start and after 24, 48, and 72 h of treatment (Supplementary Materials Section).

### 2.5. Clonogenic Assay

A clonogenic assay was performed on the LNCaP and PC-3 PCa cells treated with 1000 µg/mL of ABGE and incubated for 10 days. The results were expressed as a percentage of the number of colonies relative to the control [34] (Supplementary Materials Section).

### 2.6. Tumorsphere Formation

The tumorsphere formation assay was conducted as previously described on LNCaP and PC-3 [35,36]. A minimum of three experiments with two replicates for each condition were performed. The results are expressed as a percentage of tumorsphere area relative to the control [35] (Supplementary Materials Section).

### 2.7. Cell Migration Assay

Cell migration was assessed using a wound healing assay as previously detailed [33,35,36]. The results are presented as the percentage of the migration rate relative to the control. A minimum of three experiments with three replicates for each condition were performed. This experiment was conducted using PC-3 cell lines, but not LNCaP cells due to their lower migration capacity (Supplementary Materials Section).

### 2.8. Phosphorylation Array

Protein extracts from the LNCaP cells were collected in lysis buffer from 6-well plates after 24 h of treatment with 1000 µg/mL ABGE. The determination of protein content was conducted using a Pierce BCA Protein assay (ThermoFisher Scientific, Madrid, Spain) and adjusted with assay buffer. The data were normalized following the manufacturer’s instructions. In brief, the membranes designed for the semi-quantitative detection of 55 phosphorylated human proteins, which are part of the MAPK, AKT, JAK/STAT, and TGF-β signaling pathways, were incubated with blocking buffer for 30 min at 25 °C. The array spots’ densitometric analysis was performed using ImageJ software (version number 1.54j), with positive control spots used for normalization. The results are expressed as the log2 Fold Change in each protein signal relative to the control signal, with a log2 Fold Change of 0.2 set as the threshold [37] (Supplementary Materials Section).

### 2.9. Statistical Analysis

To calculate sample size, we performed power analysis by using G*Power 3.1.9.4 software (effect size = 0.6, α = 0.05, power = 0.85) [38]. As for the ex vivo evaluations, the experimental procedures were performed by a researcher blinded to the treatment. All experiments were conducted at least three times independently (n ≥ 3). The results from ex vivo and in vitro studies are expressed as means ± SEM. Statistical differences between the two groups were evaluated using either an unpaired parametric *t*-test or a nonparametric Mann–Whitney U test, depending on normality as determined by a Kolmogorov–Smirnov test. For comparisons involving more than two groups, a One-Way ANOVA was employed. Statistical significance was set at *p* < 0.05. All statistical analyses were performed using GraphPad Prism 9 (GraphPad Software, La Jolla, CA, USA).

## 3. Results and Discussion

In the present study, we aimed to study the potential effects of ABGE on proliferation, colony formation, tumor spheroid formation, cell migration, and the phosphorylation array in three prostate cell lines: PNT-2, LNCaP, and PC-3.

### 3.1. Ex Vivo Studies

**Figure 1:**
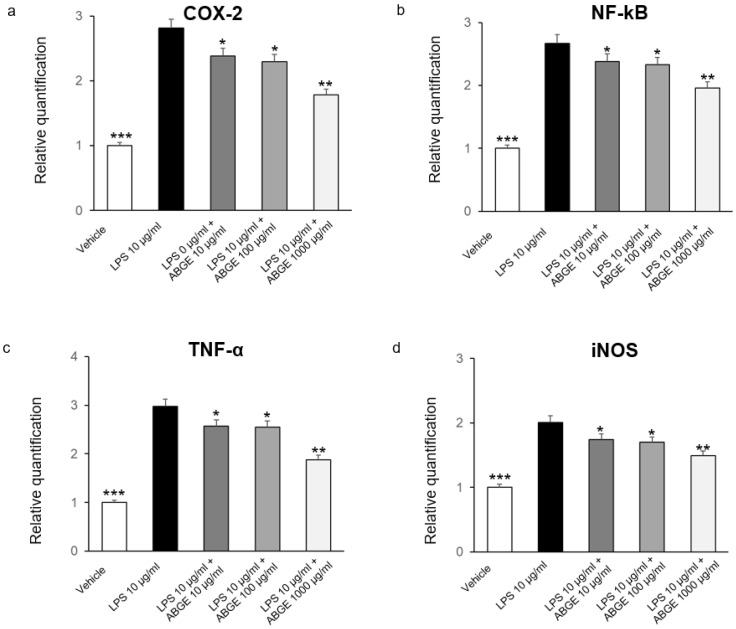
Effects of aged black garlic water extract (ABGE) (10–1000 μg/mL) on LPS-induced *cyclooxygenase* (*COX*)*-2* (**a**), *nuclear factor kappa* (*NF-κ*) *B* (**b**), *tumor necrosis factor* (*TNF)-α* (**c**), and *interleukin* (*IL)-6* (**d**) gene expression (RQ, relative quantification) in mouse prostate specimens. Data shown are means ± SEM of two independent experiments with triplicate determinations. ANOVA, * *p* < 0.05, ** *p* < 0.005; and *** *p* < 0.001 vs. LPS.

Considering the critical role of chronic inflammation in PCa, we first investigated the potential beneficial activities exerted by ABGE (10–1000 μg/mL) as a validated experimental model of inflammation [24,39]. We studied the effects of ABGE on the gene expression of pro-inflammatory mediators, including *COX-2*, *NF-kB*, *TNF-α*, and *IL-6*, on isolated LPS-stimulated prostate specimens by RT-PCR. In this context, we found that ABGE (10–1000 μg/mL) was able to inhibit gene expression of all the markers investigated, with 1000 µg/mL being the most effective dose (Figure 1a–d).

Various pro-inflammatory markers were shown to be implicated in prostatic inflammation. In this context, different phytochemicals, such as catechins, were found to modulate a number of inflammation targets, including TNF-α, COX-2, and interleukins. In the previous studies, we demonstrated that ABGE induced protective activities on colon and heart tissues treated ex vivo with LPS, which have been hypothesized to be related, at least partially, to its polyphenolic composition, with particular regards to gallic acid and catechin [24,25]. Accordingly, BenSaad et al. (2017) found that gallic acid suppressed the LPS-induced production of prostaglandin E2 and IL-6 in RAW264.7 cells [40].

Furthermore, we previously found that ABGE (1 g kg^−1^) exerted protective effects in rats in vivo [25] in a dose which could be translated to 1 g day in humans.

### 3.2. Cell Proliferation in Basal Conditions

**Figure 2:**
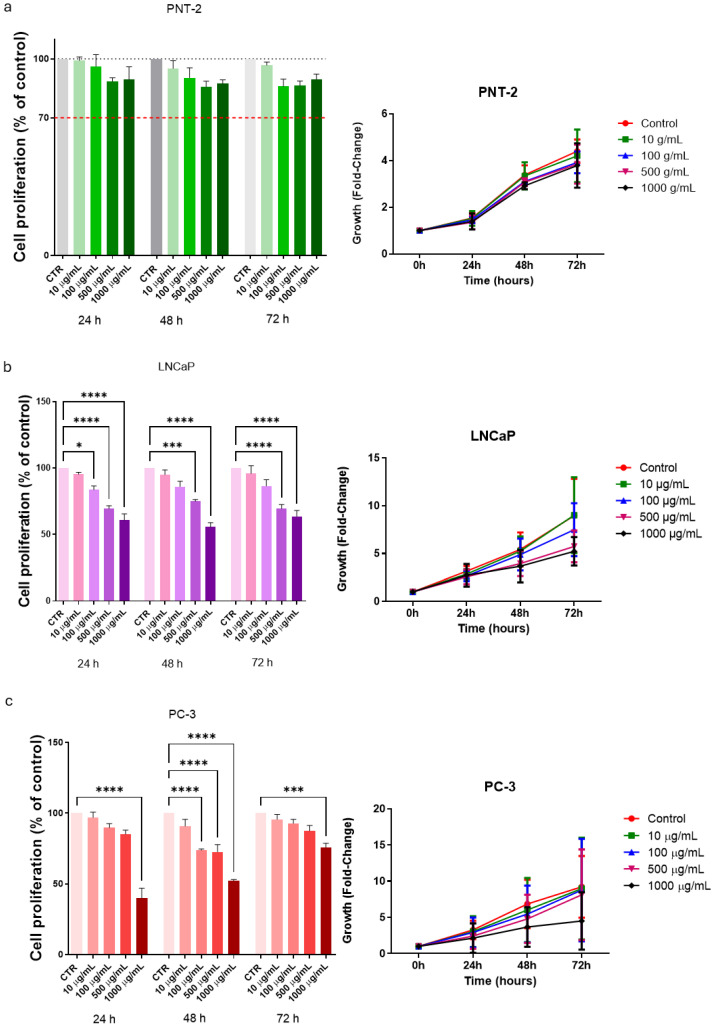
Effects of aged black garlic water extract (ABGE) on cell proliferation of control prostate (PNT-2) (**a**), androgen-dependent (LNCaP) (**b**), and androgen-independent (PC-3) (**c**) prostate cancer (PCa) cells. Cell proliferation and growth were evaluated by resazurin reagent after incubation for 24, 48, and 72 h of PNT-2, LNCaP, and PC-3 cell lines with ABGE at different concentrations (10, 100, 500, and 1000 µg/mL) or vehicle. Data shown are means ± SEM of 3 independent experiments with 3 replicates of each condition. ANOVA, * *p* < 0.05, *** *p* = 0.001, **** *p* < 0.0001 vs. vehicle.

Cell proliferation was measured after 24, 48, and 72 h of the treatment with ABGE (10, 100, 500, and 1000 µg/mL) in basal conditions. The non-tumor prostate cell line PNT-2 was used as the control cell line. In the control prostate line PNT-2, ABGE (10–1000 µg/mL) did not affect cell proliferation more compared to that of the control group at any concentration at the different times. In agreement, we previously reported that ABGE did not modify the viability of cardiomyoblast (H9c2) cells or the human fibroblast HFF-1 cell line [24,25] (Figure 2a).

The LNCaP cell line is derived from lymph node metastasis specimens of individuals with prostate cancer [41].

It retains the characteristics of prostate cancer tumor cytology as well as its early differentiation function, which represents the early androgen-dependent notable features of prostate cancer.

On the other hand, ABGE (10–1000 µg/mL) was able to significantly suppress LNCaP cell proliferation. Interestingly, the inhibitory effect on cell proliferation was dose-dependent, with a greater reduction at higher concentrations and longer exposure times (Figure 2b). Figure 2b shows inhibitory effects in cell proliferation after just 24 h, starting at a concentration of 100 µg/mL. The inhibition induced by the extract is also confirmed following 48 and 72 h of treatment at 500 and 1000 µg/mL concentrations.

The PC-3 cell line was isolated from human prostate cancer bone metastases with a low differentiation degree [42] and represents an androgen-independent prostate cancer cell with moderate metastatic potential in the absence of endogenous androgen receptors.

Similarly, the PC-3 cell line showed a significant reduction in cell proliferation following the treatment with ABGE (10–1000 µg/mL) compared to that of the control, with a greater decrease at higher concentrations (Figure 2c), thus confirming its antiproliferative activity also against androgen-independent cancer cells. After 48 h, we showed a significant decrease in cell proliferation starting from 100 µg/mL.

The mechanism underlying the inhibition of cell proliferation induced by ABGE is not yet clear. Dong and their collaborators (2014) demonstrated that an alcohol extract of ABG inhibited the growth of HT129 colon cancer cells probably by the inhibition of the PI3K/Akt pathway [22].

Additionally, Wang and their collaborators (2012) have demonstrated that aged black garlic water extract can inhibit the growth of gastric cancer cells in both in vitro and in vivo [23].

Moreover, an aged black garlic water extract showed dose-dependent apoptosis in human gastric cancer cell lines [23]. Notably, the extract in the prostate did not induce apoptosis in the LNCaP cells (Figure S1, Supplementary Materials).

Meanwhile, in vivo study highlighted the anti-cancer properties of the extract, including the inhibition of tumor growth in mice with tumors. The researchers proposed that the anti-cancer effects of the aged black garlic extract might be due to its antioxidant and immunomodulatory characteristics [23].

Multiple studies have indicated that black garlic possesses anti-tumor properties by inhibiting cell proliferation in both colon and gastric cancers. Jikihara et al. (2014) have performed an experiment using aged garlic extract on F344 rats and DLD-1 human colon cancer cells. The findings revealed antiproliferative effects in both adenoma and adenocarcinoma lesions [43].

### 3.3. Colony Formation

**Figure 3:**
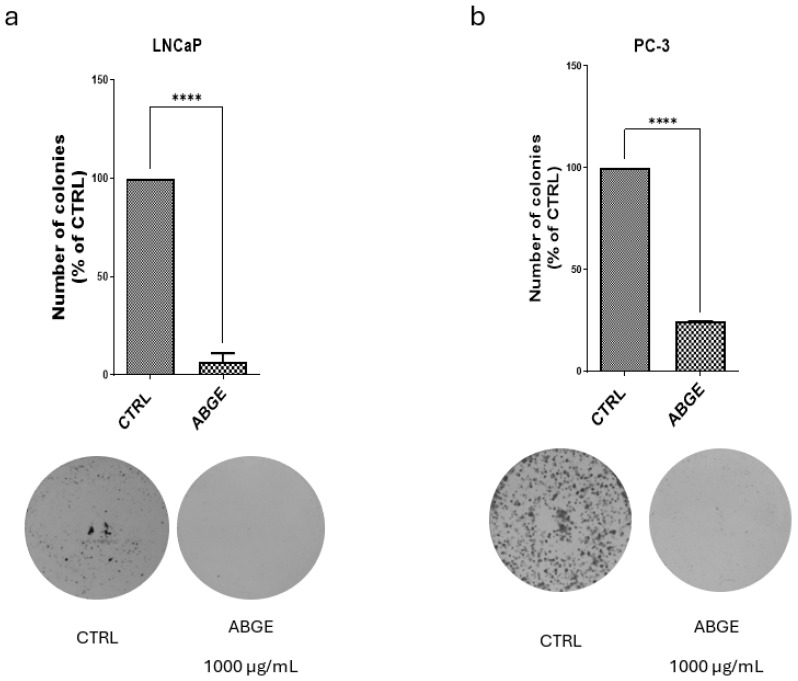
Effects of aged black garlic water extract (ABGE) on colony formation of LNCaP (**a**) and PC-3 (**b**) cell lines in response to ABGE at 1000 µg/mL or vehicle. Data shown are means ± SEM of 3 independent experiments with 3 replicates of each condition. ANOVA, **** *p* < 0.0001 vs. vehicle.

The analysis of the colony-forming ability of the LNCaP and PC-3 cell lines was performed after the treatment with ABGE (1000 µg/mL) or the vehicle. The highest concentration was chosen because it proved to be the most effective, while remaining biocompatible. In the LNCaP cell line (Figure 3a), the treatment with 1000 µg/mL of ABGE significantly reduced number of colonies formed compared to that of the control, suggesting that ABGE is effective in decreasing the long-term proliferative capacity of androgen-dependent cancer cells. Similarly, in the PC-3 cell line, the treatment with ABGE (1000 µg/mL) (Figure 3b) led to more inhibitory effects in colony formation compared to those of the control, further supporting the potential activity of the extract as an anti-tumor agent. These effects might be due to the presence of polyphenolic compounds in ABGE. In agreement, Jang et al. (2020) demonstrated that gallic acid can inhibit colony formation in various cancer cell lines [40].

### 3.4. Tumor Spheroid Formation

**Figure 4:**
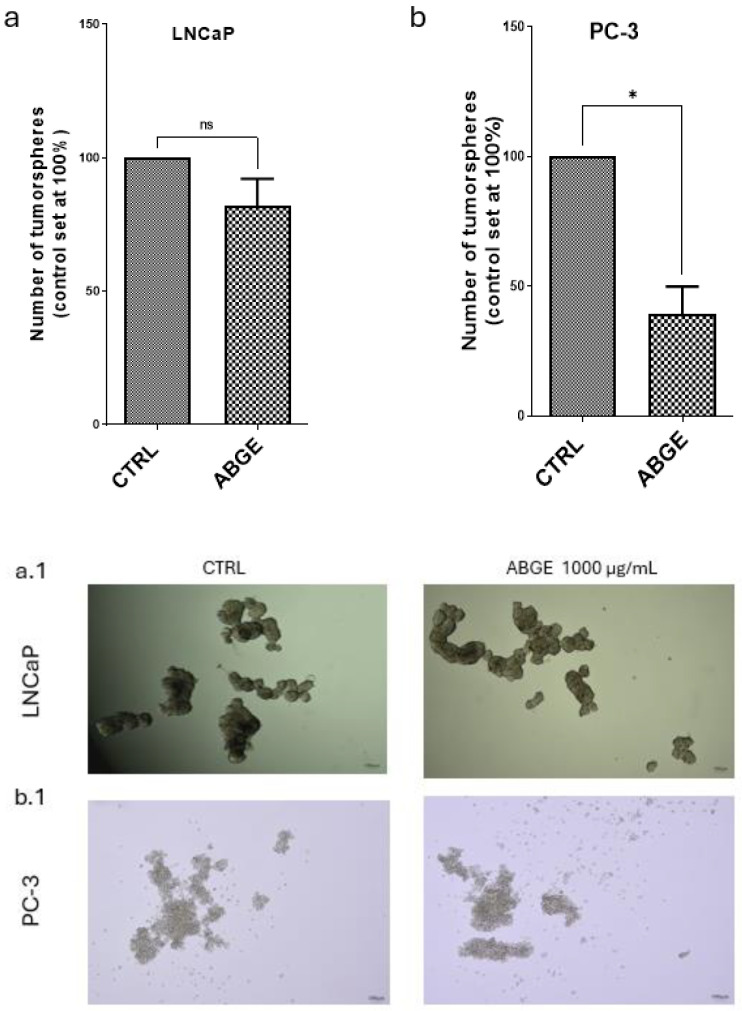
Effects of aged black garlic water extract (ABGE) on tumor spheroid formation of LNCaP (**a**,**a.1**) and PC-3 (**b**,**b.1**) cell line with ABGE at 1000 µg/mL or vehicle. Data shown are means ± SEM of 3 independent experiments with 3 replicates of each condition. ANOVA, * *p* < 0.05 vs. vehicle.

Tumor spheroid formation was assessed by measuring the number of spheroids after the treatment with ABGE (1000 µg/mL) or the vehicle in the LNCaP and PC-3 cell lines. In the LNCaP cells (Figure 4a), the treatment with ABGE (1000 µg/mL) did not significantly affect the number of spheroids, which remained unchanged compared to that of the vehicle. However, our present findings also showed that in the PC-3 cells (Figure 4b), ABGE (1000 µg/mL) significantly reduced the number of spheroids compared to that of the control, suggesting that ABGE impedes the proliferation of cancer cells.

### 3.5. Migration Assay

**Figure 5:**
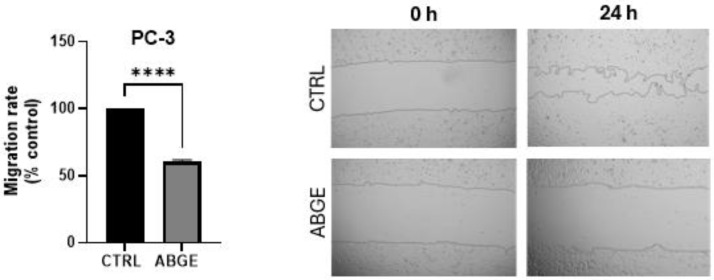
Effects of aged black garlic water extract (ABGE) on migration of PC-3 cell line with ABGE at 1000 µg/mL or vehicle for 24 h. Data shown are means ± SEM of 3 independent experiments with 3 replicates of each condition. ANOVA, **** *p* < 0.0001 vs. vehicle.

The cell migration assay was conducted only on the PC-3 cell line because the morphology of LNCaP cells does not allow for accurate migration assessment. The treatment with 1000 µg/mL of ABGE more significantly reduced the cell migration rate compared to that of the control after 24 h of incubation. The reduction in cell migration in PC-3 (Figure 5) suggests that ABGE may also limit the capacity of cancer cells to spread. In agreement, recently, ABG (dissolved in 0.9% normal saline) extract was found able to impede cell migration in breast cancer cells [44].

### 3.6. Cell Proliferation after LPS Pre-Treatment

**Figure 6:**
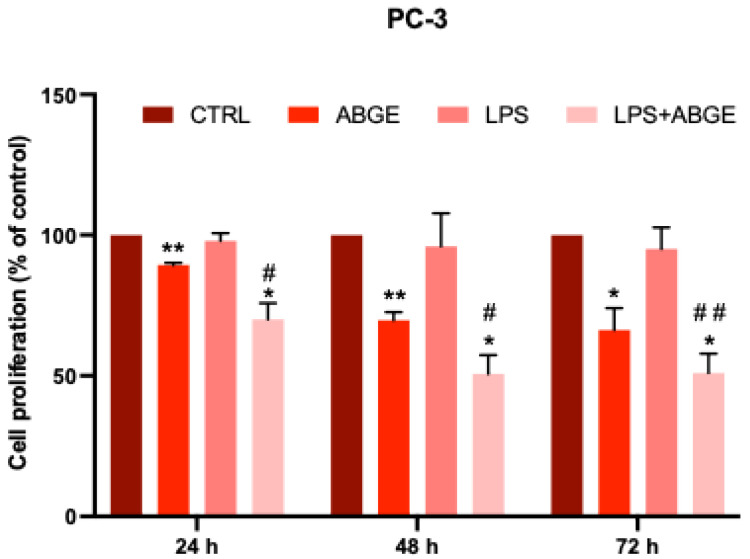
Effects of aged black garlic water extract (ABGE) in combination to LPS on cell proliferation of androgen-independent PC-3 cells. Cell proliferation was evaluated by resazurin reagent after incubation for 24, 48, and 72 h of PC-3 cell line with ABGE at 1000 µg/mL or vehicle. Data shown are means ± SEM of 3 independent experiments with 3 replicates of each condition. ANOVA, * *p* < 0.05, ** *p* < 0.005 vs. vehicle. ANOVA, # *p* < 0.05, ## *p* < 0.005 vs. ABGE.

Considering the previously found effects of ABGE into a pro-inflammatory cell context [18], we then decided to evaluate its potential interaction with LPS. We studied the effects of ABGE (10–1000 µg/mL) on LPS-treated cell proliferation in the PC-3 line (Figure 6), which was chosen for its higher aggressiveness compared to that of the LNCaP cell line, as supported by previous studies [45]. In this context, Xu and their collaborators (2021) showed that LPS combined with ATP significantly increased the proliferation and migration of PC-3 cells, reducing apoptosis. This effect was related to the stimulation of the NLRP3/caspase-1 inflammasome, hypothesizing that inflammation plays a crucial role in prostate cancer progression [8]. Interestingly, our data indicate that the LPS pre-treatment may sensitize the PCa cells to ABGE (Figure 6).

Gallic acid and catechin, which are the main components of ABGE, as previously shown [24,25], are well known to suppress, proliferate, and stimulate the apoptosis of PCa cells [39,40].

Moreover, a previous study showed that gallic acid decreased the viability of PCa cell lines, but not normal cells’ viability [46]. In agreement, we could suggest the potential involvement of polyphenolic compounds, with particular regards to gallic acid and catechin, due to the beneficial effects induced by ABGE on PCa proliferation.

### 3.7. Phosphorylation Array

A wide body of evidence shows that the MAPK, AKT, JAK/STAT, and TGF-β pathways play a key role in cell proliferation, survival, apoptosis, and growth [23,39,40,41]. In our study, we analyzed the phosphorylation of the key proteins that participate in these signaling pathways in response to the treatment with ABGE (1000 µg/mL) or the vehicle using a phosphorylation array.

**Figure 7:**
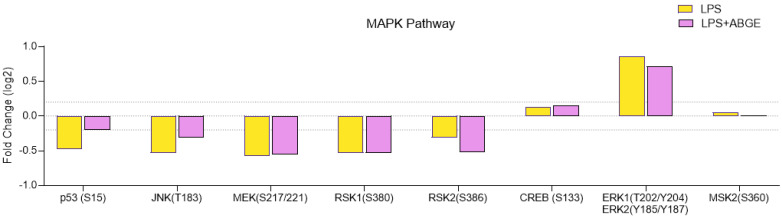
The MAPK signaling pathway in the phosphoprotein array in response to 24 h treatment of 1000 µg/mL ABGE. The log2 Fold Change in the phosphorylation protein level in comparison with that for the control condition (threshold: log2 Fold Change = 0.2).

Specifically, the MAPK signaling pathway showed significant modulation in response to the treatment with ABGE (Figure 7). Proteins such as ERK1/2 and JNK showed reduced phosphorylation, suggesting that ABGE (1000 µg/mL) could inhibit these signaling pathways. In this context, the MAPK signaling pathway is critically related to cell proliferation and survival [47]. Furthermore, the reduced phosphorylation of ERK1/2 (T202/Y204) and JNK (T183/Y185) suggests reduced cell proliferation and the potential inhibition of the apoptotic response [48].

p53 is involved in regulation of cell growth, DNA repair, survival, cycle, autophagy, senescence, and apoptosis [49,50].

After the injection of knockdown of ribosomal S6 protein kinases (RSK) 1 and RSK2 in mouse femurs, there was a reduction in osteolytic lesions in the PC3 cells compared to those in the control cells [51].

In our present study, we found that ABGE increased the quantity of LPS-treated p53, while decreased the RSK2 phosphorylation levels (Figure 7), which could be related to the anti-cancer properties of the extract.

**Figure 8:**
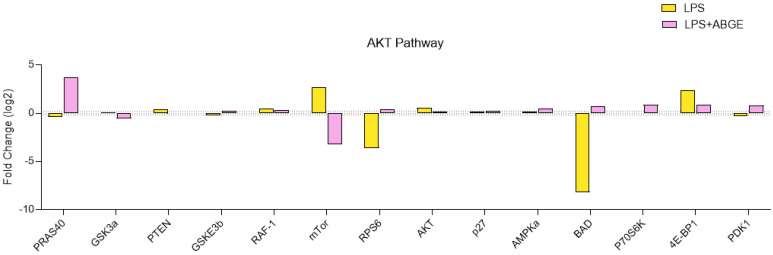
The AKT signaling pathway in the phosphoprotein array in response to 24 h treatment of 1000 µg/mL ABGE. The log2 Fold Change in the phosphorylation protein level in comparison with that for the control condition (threshold: log2 Fold Change = 0.2).

The role of AKT signaling pathway in cell survival and growth is also well known [23]. ABGE (1000 µg/mL) showed variable effects on the phosphorylation of both the AKT and downstream proteins (Figure 8). 

The reduced phosphorylation of mTOR suggests decreased protein synthesis and cell growth [52]. mTOR activation has been shown to induce the phosphorylation of many substrates, such as eukaryotic translation initiation factor 4E (eIF4E)-binding proteins (4E-BP1), and mTOR kinase inhibitors have been reported to block p4E-BP1 [53].

Our findings show that ABGE reduced the LPS-treated mTOR and 4E-BP1 phosphorylation levels. In agreement, considering that high levels of 4E-BP1 have been measured in prostate cancer cells, we can speculate that mTOR and 4E-BP1 could be involved, at least in part, in the beneficial effects induced by ABGE [54].

Moreover, ABGE also lowers the LPS-treated levels of glycogen synthase kinase (GSK)-3, phosphatase, tensin-homolog in chromosome 10 (PTEN), and serine/threonine kinase Raf-1 (RAF-1), which are involved in cancer development and progression [55,56,57].

On the other hand, ABGE increased the LPS-treated levels of p27 and AMP-activated protein kinase (AMPK), which possess a well-known suppressor role in carcinogenesis [58,59].

The JAK/STAT signaling pathway plays a key role in numerous essential biological processes, such as differentiation, cell proliferation, immune regulation, and apoptosis [48].

The inactivation of Src induced a reduction in the migration and growth in PCa cell lines [60,61]. 

**Figure 9:**
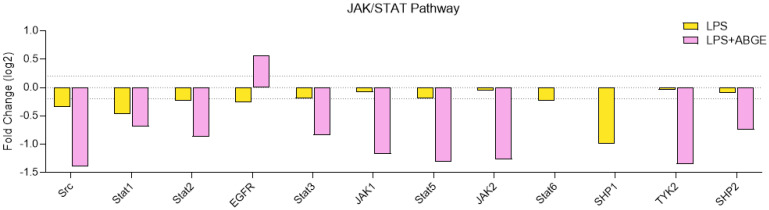
The JAK/STAT signaling pathway in the phosphoprotein array in response to 24 h treatment of 1000 µg/mL ABGE. The log2 Fold Change in the phosphorylation protein level in comparison with that for the control condition (threshold: log2 Fold Change = 0.2).

In addition, the reduced phosphorylation of STAT1, STAT2, STAT3, and STAT5 (Figure 9) indicates a potential decrease in proliferative signaling and cancer development [62,63,64,65].

Moreover, the decreased phosphorylation of JAK1 and JAK2 suggests decreased signal transduction promoting prostate cancer cell proliferation and survival [66,67].

In agreement, TYK2 signaling promotes the invasiveness of prostate cancer cells [68].

Accordingly, the involvement of SHP2 in several cancer-related processes has been reported [69]. 

Actually, our findings, showing that ABGE decreased the LPS-treated Src, STAT1, STAT2, STAT3, STAT5, JAK1, JAK 2, TYK2, and SHP2 phosphorylation levels, could suggest the potential protective role of the extract in PC-3 cells.

**Figure 10:**
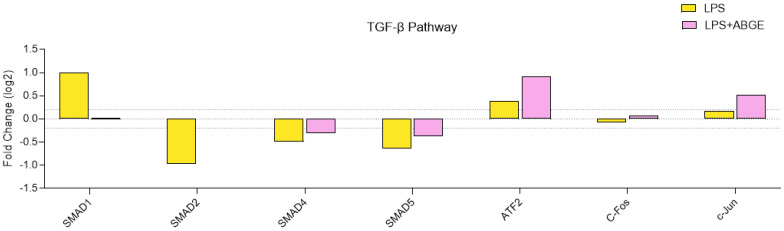
The TGF-β pathway in the phosphoprotein array in response to 24 h treatment of 1000 µg/mL ABGE. The log2 Fold Change in the phosphorylation protein level in comparison with that for the control condition (threshold: log2 Fold Change = 0.2).

Regarding the TGF-β pathway (Figure 10) involved in cell growth regulation and tumor progression [49], we showed that ABGE (1000 µg/mL) decreased the phosphorylation of SMAD1, suggesting reduced TGF-β signaling, which may be associated with reduced cell invasiveness [70].

The previous studies reported a correlation between SMAD2 and SMAD4, which are involved in the inhibition of cell growth [71].

ATF2 has been found as a tumor promoter in various human cancers, such as prostate cancer [72].

Furthermore, c-Jun or c-Fos overexpression has been directly related with PCa cell line invasiveness, and the phosphorylated c-Jun levels are high in PCa [73].

In our study, we showed that ABGE decreased the quantity of LPS-treated SMAD1, while it increased the SMAD2, SMAD4, AFT2, c-Jun, and c-Fos phosphorylation levels, further confirming the potential protective role of the extract.

In the literature, there are not many studies about the effects of ABGE on the prostate; thus, its mechanisms are not particularly well known.

## 4. Conclusions

In conclusion, our results showed the potential anti-inflammatory and anti-proliferative effects of ABGE on prostate cancer. In this context, ABGE reduced the gene expression of the different biomarkers involved in inflammatory response, such as *COX-2*, *TNF-α*, *IL-6*, and *NF-kB*, also modulating relevant signaling pathways, including AKT, MAPK, TGF-β, and JAK/STAT. Furthermore, we performed different in vitro assays, where ABGE had beneficial effects on both the prostate cancer lines. Therefore, our results suggest that ABGE might be potentially used as a diet supplement for health promotion and a source of bio-organic compounds with antitumor properties in PCa.

A limitation of our study is that we have not evaluated specific targeting, as well as the signaling pathways modulating the potential anti-inflammatory and anti-cancer effects of the extract. However, further studies are needed in the future to accurately evaluate the in vivo activity of ABGE in reducing inflammation and cancer, as well as its potential negative effects on the body.

## Data Availability

The original contributions presented in the study are included in the article and Supplementary Materials, further inquiries can be directed to the corresponding authors request.

## Supplementary Materials

The following supporting information can be downloaded at: https://www.mdpi.com/article/10.3390/nu16173025/s1. Figure S1: Apoptosis induction in response to ABGE (1000 µg/mL) in LNCaP cells. Annexin V positive cells were considered as apoptotic. Annexin positivity was determined by flow cytometry (LSRFortessa SORP) with FITC Annexin V Apoptosis Detection Kit I (BD Pharmingen, San Jose, CA, USA; #556547) at 10 μg/mL in PBS. References [24,27,28,29,31,32,33,34,35,36,37,38] are cited in the supplementary materials.
