# Supplementary material for: The Protective Effects of an Aged Black Garlic Water Extract on the Prostate
Source: Nutrients. 2024 Sep 7;16(17):3025. doi: 10.3390/nu16173025 (PMC11396974; doi:10.3390/nu16173025)
Supplement: Supplementary file 1 [file nutrients-16-03025-s001.zip › nutrients-3151672-supplementary.pdf]

nutrients

MDPI

Supplementary Materials

The Protective Effects of an Aged Black Garlic Water Extract on the Prostate

Maria Loreta Libero [1,2,3,†], Antonio J. Montero-Hidalgo [2,3,4,5,†], Lucia Recinella [1,*], Raúl M. Luque [2,3,4,5,*], Daniele Generali [6,7], Alessandra Acquaviva [1], Giustino Orlando [1], Claudio Ferrante [1], Luigi Menghini [1], Simonetta Cristina Di Simone [1], Nilofar Nilofar [1], Annalisa Chiavaroli [1], Luigi Brunetti [1] and Sheila Leone [1]

1 Department of Pharmacy, "G. d'Annunzio" University, 66013 Chieti, Italy; maria.libero@unich.it (M.L.L.); alessandra.acquaviva@unich.it (A.A.); giustino.orlando@unich.it (G.O.); claudio.ferrante@unich.it (C.F.); luigi.menghini@unich.it (L.M.); simonetta.disimone@unich.it (S.C.D.S.); nilofar.nilofar@unich.it (N.N.); annalisa.chiavaroli@unich.it (A.C.); luigi.brunetti@unich.it (L.B.); sheila.leone@unich.it (S.L.)
2 Department of Cell Biology, Physiology and Immunology, University of Cordoba, 14014 Cordoba, Spain; b42mohia@uco.es
3 Maimonides Biomedical Research Institute of Cordoba (IMIBIC), 14004 Cordoba, Spain
4 Reina Sofia University Hospital (HURS), 14004 Cordoba, Spain
5 Centro de Investigación Biomédica en Red de Fisiopatología de la Obesidad y Nutrición (CIBERobn), 14004 Cordoba, Spain
6 Department of Medical, Surgical and Health Sciences, University of Trieste, 34149 Trieste, Italy; dgenerali@units.it
7 Department of Advanced Translational Microbiology, Institute for Maternal and Child Health-IRCCS "Burlo Garofolo", 34137 Trieste, Italy
* Correspondence: lucia.recinella@unich.it (L.R.); raul.luque@uco.es (R.M.L.); Tel.: +39-0871-3554754 (L.R.); +34-957-213740 (R.M.L.)
† These authors contributed equally to this work.

Abstract: Chronic inflammation is a recognized risk factor for various cancers, including prostate cancer (PCa). We aim to explore the potential protective effects of aged black garlic extract (ABGE) against inflammation-induced prostate damage and its impact on prostate cancer cell lines. We used an ex vivo model of inflammation induced by Escherichia coli lipopolysaccharide (LPS) on C57BL/6 male mouse prostate specimens to investigate the anti-inflammatory properties of ABGE. Gene expression levels of pro-inflammatory biomarkers (*COX-2*, *NF-κB*, *TNF-α*, *IL-6*) were measured. Additionally, we evaluated ABGE's therapeutic effects on prostate cancer cell lines through in vitro functional assays, including colony formation, tumorsphere formation, migration assays, and phosphorylation arrays to assess signaling pathways (MAPK, AKT, JAK/STAT, TGF-β). ABGE demonstrated significant anti-inflammatory and antioxidant effects in preclinical models, partly attributed to its polyphenolic content, notably catechin and gallic acid. In the ex vivo model, ABGE reduced gene expression levels of *COX-2*, *NF-κB*, *TNF-α*, and *IL-6*. In vitro studies showed that ABGE inhibited cell proliferation, induced apoptosis, and modulated the cell cycle in prostate cancer cells, suggesting its potential as a therapeutic agent. ABGE exhibits promising anti-inflammatory and anti-cancer properties, supporting further investigation into ABGE as a potential agent for managing inflammation and prostate cancer.

Keywords: ABGE; prostate; inflammation; cancer

1. Materials and Methods

1.1. Extraction and Sample Preparation of ABGE

ABG cloves were provided in dried form by Il Grappolo S.r.l. (Soliera, Modena, Italy). 10 grams of garlic cloves were manually crushed using a garlic press, and the resulting juice and debris were collected in a centrifuge tube by rinsing the crusher with 10 mL of

water. After shaking the tube 10 times, it was placed in a Trans-sonic T460 ultrasonic bath from Elma (Singen, Germany) for 15 minutes at room temperature. Subsequently, the mixture was centrifuged twice at 4000 rpm for 10 minutes each [1-3]. The supernatant was then filtered and stored until further analysis.

1.2. Ex Vivo Studies

Adult C57BL/6 male mice (3-month-old, weight 20–25 g, n = 25) were housed in Plexiglas cages (2–4 animals per cage; 55 cm x 33 cm x 19 cm) and maintained under standard laboratory conditions (21 ± 2 °C; 55 ± 5% humidity) on a 14/10 h light/dark cycle, with ad libitum access to water and food. Housing conditions and experimentation procedures were strictly in agreement with the European Community ethical regulations (EU Directive no. 26/2014) on the care of animals for scientific research. In agreement with the recognized principles of "Replacement, Refinement and Reduction in Animals in Research", prostate specimens were obtained as residual material from vehicle-treated mice randomized in our previous experiments, approved by local ethical committee ('G. d'Annunzio' University, Chieti, Italy) and Italian Health Ministry (Project no. 885/2018-PR).

Mice sacrifice was performed by CO2 inhalation (100% CO2 at a flow rate of 20% of the chamber volume per min). After collection, isolated prostate specimens were maintained in a humidified incubator with 5% CO2 at 37 °C for 4 h (incubation period), in RPMI buffer with added bacterial LPS (10 μg/mL), as previously described [3-4]. During the incubation period, the tissues were challenged with scalar concentrations of ABGE (10-1000 μg/ml) or vehicle (controls) (n = 5 for each experimental group).

Total RNA extraction from the single prostate specimen was performed using TRI Reagent (Sigma–Aldrich, St. Louis, MO, USA), in agreement with the manufacturer's protocol. One microgram of total RNA extracted from each sample in a 20 μL reaction volume was reverse transcribed using High Capacity cDNA Reverse Transcription Kit (Thermo Fisher Scientific Inc., Monza, Italy). Reactions were incubated in a 2720 Thermal Cycler (Thermo Fisher Scientific Inc., Monza, Italy) initially at 25 °C for 10 min, then at 37 °C for 120 min, and finally at 85 °C for 5 s. Gene expression of *COX-2*, *NF-κB*, *TNF-α*, and *iNOS* was measured by quantitative real-time PCR using TaqMan probe-based chemistry, as previously described [5-6]. PCR primers and TaqMan probes, including *β-actin* used as the housekeeping gene, were purchased from Thermo Fisher Scientific Inc. (Assays-on-Demand Gene Expression Products, Mm00478374_m1 for *COX-2* gene, Mm00443258_m1 for *TNF-α* gene, Mm00476361_m1 for *NF-κB* gene, Mm00446190_m1 for *IL-6* gene, Mm00607939_s1 for *β-actin* gene). The real-time PCR was carried out in triplicate for each cDNA sample in relation to each of the investigated genes. Data were elaborated with the Sequence Detection System (SDS) software version 2.3 (Thermo Fisher Scientific Inc.). Relative quantification of gene expression was performed by the comparative $2^{-\Delta\Delta Ct}$ method [7].

1.3. Cell Culture

Cell lines from control prostate (PNT-2), androgen-dependent PCa (LNCaP), and androgen-independent PCa (PC-3) were sourced from the American Type Culture Collection (Manassas, VA, USA) and maintained in a humidified incubator with 5% CO2 at 37 °C, following the manufacturer's guidelines as previously outlined [8-9]. To verify the authenticity of these cell lines, short tandem repeat (STR) sequence analysis was conducted using the GenePrint 10 System (Promega, Barcelona, Spain), and the absence of mycoplasma contamination was confirmed by PCR, as previously described [8].

1.4. Cell Proliferation

Cell proliferation was evaluated using resazurin reagent (Canvax Biotech, Cordoba, Spain), as previously described [8]. In brief, 3000 to 5000 cells were plated per well in 96-

well plates, at 5% FBS and then treated with varying concentrations (10–1000 μg/mL) of ABGE or vehicle for 24, 48, and 72 hours. Cell proliferation was measured at the start and after 24, 48, and 72 hours of treatment by recording fluorescence intensity (560 nm excitation and 590 nm emission) following a 3-hour incubation with 10% resazurin using the FlexStation III system (Molecular Devices, Sunnyvale, CA, USA). The results were expressed as a percentage of fluorescence intensity relative to the control (vehicle-treated). A minimum of three experiments with three replicates for each condition were conducted.

1.5. Clonogenic Assay

To evaluate the clonogenic capacity of LNCaP and PC-3 PCa cells, 2000 cells were seeded into 6-well plates, treated with 1000 μg/mL of ABGE, and incubated for 10 days. Following the incubation period, the medium was removed, and the colonies were washed with PBS, stained with a crystal violet solution (6% glutaraldehyde, 0.5% crystal violet) for 30 minutes, then rinsed and allowed to air dry. A minimum of three experiments with two replicates for each condition were conducted. The number of colonies was counted using ImageJ software (National Institutes of Health, Bethesda, MD, USA). The results were expressed as a percentage of the number of colonies relative to the control [8].

1.6. Tumorsphere Formation

The tumorsphere formation assay was conducted as previously described [9-10]. Briefly, 2000 cells per well were seeded in Corning Costar 24-well ultra-low attachment plates (Merck, Madrid, Spain) with DMEM F-12 medium supplemented with 20 ng/mL EGF (Sigma-Aldrich, Madrid, Spain). After 24 hours, 1000 μg/mL ABGE was added. The area of tumorspheres was measured after 14 days of incubation using ImageJ software. A minimum of three experiments with two replicates for each condition were conducted. Results were expressed as a percentage of tumorsphere area relative to the control [9].

1.7. Cell Migration Assay

Cell migration was assessed using a wound healing assay as previously detailed [7, 9-10]. In summary, 50,000 cells were seeded in 96-well plates and cultured until they reached confluence. Wounds were created using the IncuCyte WoundMaker (Essen BioScience, Ann Arbor, MI, USA) following the manufacturer's protocol. The wells were then rinsed with PBS and the cells were treated overnight with 1000 μg/mL of ABGE in a serum-free medium. Images of the wounds were captured immediately after wounding (0 h) and after 24 hours of treatment. Wound healing was quantified as the ratio of the scratched areas at the initial and final timepoints, analyzed using ImageJ software (National Institutes of Health, Bethesda, MD, USA). Results were presented as the percentage of the migration rate relative to the control. A minimum of three experiments with three replicates for each condition were performed. This experiment was conducted using PC-3 cell lines but not LNCaP cells due to their lower migration capacity.

1.8. Phosphorylation Array

Protein extracts from LNCaP cells were collected in lysis buffer from 6-well plates after 24 hours of treatment with 1000 μg/mL ABGE. A phosphorylation pathway profiling array was conducted using the Human Phosphorylation Pathway Profiling Array C55 kit #AAH-PPP-1–8, according to the manufacturer's instructions (Raybiotech, Inc., Peachtree Corners, GA, USA). Protein content was determined by Pierce BCA Protein assay (ThermoFisher Scientific) and adjusted with assay buffer. Data were normalized following manufacturer's instructions. Briefly, membranes designed for the semi-quantitative detection of 55 phosphorylated human proteins, which are part of the MAPK, AKT, JAK/STAT, and TGF-β signaling pathways, were incubated with blocking buffer for 30 minutes at 25 °C. Subsequently, the membranes were incubated overnight at 4 °C with 1

mL of a 4-fold dilution of pooled LNCaP cell lysates (n = 3). After washing, the membranes were treated with a detection antibody cocktail for 2 hours at 25 °C, followed by an incubation with horseradish peroxidase (HRP)-labeled anti-rabbit secondary antibody for another 2 hours at 25 °C. Signals were detected using an ECL reagent with a BioRad Universal Hood II chemiluminescence detection system (BioRad Laboratories, Hercules, CA, USA). The array spots' densitometric analysis was performed using ImageJ software, with positive control spots used for normalization. Results were expressed as the log2 fold change of each protein signal relative to the control signal, with a log2 fold change of 0.2 set as the threshold [11].

1.9. Statistical Analysis

To calculate sample size, we performed a power analysis by using G*Power 3.1.9.4 software (effect size = 0.6, α=0.05, power = 0.85) [12]. As for ex vivo evaluations, the experimental procedures were performed by a researcher blinded to the treatment. All experiments were conducted at least three times independently ($n \geq 3$). Results from ex vivo and in vivo studies were expressed as means ± SEM. Statistical differences between two groups were evaluated using either an unpaired parametric t-test or a nonparametric Mann–Whitney U test, depending on normality as determined by the Kolmogorov–Smirnov test. For comparisons involving more than two groups, a One-Way ANOVA was employed. Statistical significance was set at $p < 0.05$. All statistical analyses were performed using GraphPad Prism 9 (GraphPad Software, La Jolla, CA, USA).

2. Results

2.1. Apoptosis Assay

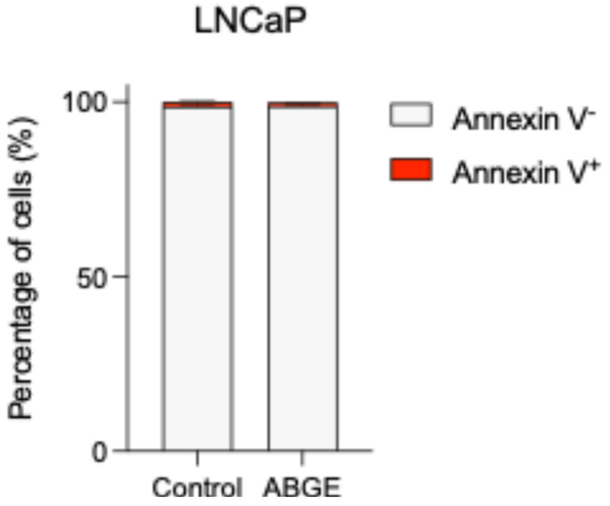

Figure S1. Apoptosis induction in response to ABGE (1000 µg/mL) in LNCaP cells. Annexin V positive cells were considered as apoptotic. Annexin positivity was determined by flow cytometry (LSRFortessa SORP) with FITC Annexin V Apoptosis Detection Kit I (BD Pharmingen, San Jose, CA; #556547) at 10 µg/mL in PBS.

References

1. Recinella, L.; Libero, M.L.; Citi, V.; Chiavaroli, A.; Martelli, A.; Foligni, R.; Mannozzi, C.; Acquaviva, A.; Di Simone, S.; Calderone, V.; et al. Anti-Inflammatory and Vasorelaxant Effects Induced by an Aqueous Aged Black Garlic Extract Supplemented with Vitamins D, C, and B12 on Cardiovascular System. *Foods* **2023**, 12, 1558, doi:10.3390/foods12071558.
2. Fujisawa, H.; Suma, K.; Origuchi, K.; Seki, T.; Ariga, T. Thermostability of Allicin Determined by Chemical and Biological Assays. *Biosci Biotechnol Biochem* **2008**, 72, 2877–2883, doi:10.1271/bbb.80381.
3. Fujisawa, H.; Suma, K.; Origuchi, K.; Kumagai, H.; Seki, T.; Ariga, T. Biological and Chemical Stability of Garlic-Derived Allicin. *J Agric Food Chem* **2008**, 56, 4229–4235, doi:10.1021/jf8000907.

4. Del Rio-Moreno, M.; Alors-Perez, E.; Borges de Souza, P.; Prados-Gonzalez, M.E.; CastaÑo, J.P.; Luque, R.M.; Gahete, M.D. Peptides Derived from the Extracellular Domain of the Somatostatin Receptor Splicing Variant SST5TMD4 Increase Malignancy in Multiple Cancer Cell Types. *Transl Res* **2019**, 211, 147–160, doi:10.1016/j.trsl.2019.02.013.
5. Recinella, L.; Micheli, L.; Chiavaroli, A.; Libero, M.L.; Orlando, G.; Menghini, L.; Acquaviva, A.; Di Simone, S.; Ferrante, C.; Ghelardini, C.; et al. Anti-Inflammatory Effects Induced by a Polyphenolic Granular Complex from Olive (Olea Europaea, Mainly Cultivar Coratina): Results from In Vivo and Ex Vivo Studies in a Model of Inflammation and MIA-Induced Osteoarthritis. *Nutrients* **2022**, 14, 1487, doi:10.3390/nu14071487.
6. Recinella, L.; Libero, M.L.; Veschi, S.; Piro, A.; Marconi, G.D.; Diomede, F.; Chiavaroli, A.; Orlando, G.; Ferrante, C.; Florio, R.; et al. Effects of GHRH Deficiency and GHRH Antagonism on Emotional Disorders in Mice. *Cells* **2023**, 12, 2615, doi:10.3390/cells12222615.
7. Livak, K.J.; Schmittgen, T.D. Analysis of Relative Gene Expression Data Using Real-Time Quantitative PCR and the 2(-Delta Delta C(T)) Method. *Methods* **2001**, 25, 402–408, doi:10.1006/meth.2001.1262.
8. Hormaechea-Agulla, D.; Gahete, M.D.; Jiménez-Vacas, J.M.; Gómez-Gómez, E.; Ibáñez-Costa, A.; L-López, F.; Rivero-Cortés, E.; Sarmento-Cabral, A.; Valero-Rosa, J.; Carrasco-Valiente, J.; et al. The Oncogenic Role of the In1-Ghrelin Splicing Variant in Prostate Cancer Aggressiveness. *Mol Cancer* **2017**, 16, 146, doi:10.1186/s12943-017-0713-9.
9. Fuentes-Fayos, A.C.; G-García, M.E.; Pérez-Gómez, J.M.; Montero-Hidalgo, A.J.; Martín-Colom, J.; Doval-Rosa, C.; Blanco-Acevedo, C.; Torres, E.; Toledano-Delgado, Á.; Sánchez-Sánchez, R.; et al. Metformin and Simvastatin Exert Additive Antitumour Effects in Glioblastoma via Senescence-State: Clinical and Translational Evidence. *eBioMedicine* **2023**, 90, 104484, doi:10.1016/j.ebiom.2023.104484.
10. Jiménez-Vacas, J.M.; Herrero-Aguayo, V.; Gómez-Gómez, E.; León-González, A.J.; Sáez-Martínez, P.; Alors-Pérez, E.; Fuentes-Fayos, A.C.; Martínez-López, A.; Sánchez-Sánchez, R.; González-Serrano, T.; et al. Spliceosome Component SF3B1 as Novel Prognostic Biomarker and Therapeutic Target for Prostate Cancer. *Transl Res* **2019**, 212, 89–103, doi:10.1016/j.trsl.2019.07.001
11. León-González, A.J.; Sáez-Martínez, P.; Jiménez-Vacas, J.M.; Herrero-Aguayo, V.; Montero-Hidalgo, A.J.; Gómez-Gómez, E.; Madrona, A.; Castaño, J.P.; Espartero, J.L.; Gahete, M.D.; et al. Comparative Cytotoxic Activity of Hydroxytyrosol and Its Semisynthetic Lipophilic Derivatives in Prostate Cancer Cells. *Antioxidants (Basel)* **2021**, 10, 1348, doi:10.3390/antiox10091348.
12. Kang, H. Sample Size Determination and Power Analysis Using the G*Power Software. *J Educ Eval Health Prof* **2021**, *18*, 17, doi:10.3352/jeehp.2021.18.17.

Disclaimer/Publisher's Note: The statements, opinions and data contained in all publications are solely those of the individual author(s) and contributor(s) and not of MDPI and/or the editor(s). MDPI and/or the editor(s) disclaim responsibility for any injury to people or property resulting from any ideas, methods, instructions or products referred to in the content.
